# The Dentist at Thirty-Five

**Published:** 1920-09

**Authors:** George Wood Clapp

**Affiliations:** New York, N. Y.


### THE DENTIST AT THIRTY-FIVE

BY GEORGE WOOD CLAPP, D.D.S., NEW YORK, N. Y.

Eight dentists in every ten who live to be thirty-five years of age will live to be fifty-five years old. And those living at the age of fifty-five will average to live to be seventy-two. From the age of thirty-five to the age of fifty-five these dentists should earn more than their expenses. Then, if ever, their earnings must be enough greater than their expenditures to recompense the cost of their education and to provide a surplus of money which will support them after they become unable to earn more than their expenses. With rare exceptions, dentists will have acquired no surplus up to the age of thirty-five, and will be unable to acquire one after fifty-five.

From the age of fifty-five to the age of sixty-five, the average dentist will earn no more than the amount of his necessary expenses. After the age of sixty-five, he will be increasingly dependent upon the surplus accumulated before the age of thirty-five. If he does not have such a surplus, he will be in the position of 97 per cent. of the American people, who, after sixty-five, are dependent upon friends or charity for the necessities of life.

It behooves any dentist at the age of thirty-five, however exceptional he may consider himself, to recognize that these figures have been obtained by averaging a great number of lives, and that the percentage of error is very small. As many exceptional lives would probably be included in the number averaged, the dentist's life is very likely, before all these years have passed, to conform closely to the average. In any event, he will be safer to plan his conduct on these averages than on any individual conviction of his own superiority to the average.

Unfortunately for us all, we have been such poor business men in the past, that the clearly defined lines which should be available for the guidance of the dentist at thirty-five are not as well established as they should be. But some facts are beginning to be clearly seen. They are fundamental and of the greatest economic importance.

Before outlining these facts, a word of explanation may be advisable. In studying nearly everything connected with the practice of dentistry, it is necessary to set a standard of life and service, and this must be done here. The figures apply with greatest force to the dentist who seeks to live a life well balanced between work and recreation, so that his physical vigor is well maintained; and who seeks to keep sufficiently abreast of the advances of his profession to be able to render a good quality of service—at least in the forms of dentistry he practices. It is by no means certain that they do not apply equally to dentists who work unceasingly, with no attention to recreation, but this application is not so well established.

In the well-balanced life, it is doubtful whether the dentist can average more than two thousand office hours for each of the years between the ages of thirty-five and fifty-five. It is growing increasingly clear that not more than one thousand of these hours, yearly, will be income hours. In offices where careful records have been kept for several years, the average is about nine hundred and fifty income hours per year per dentist.

The dentist, then, at the age of thirty-five years, has for sale one thousand income hours per year for twenty years, twenty thousand income hours in all. From the sale of these hours he must recoup an expense for special education and office equipment of about five thousand dollars; must meet all his living expenses, including the education of any children and any luxuries by which he may be beguiled, and lay aside, in the form of income-producing securities, enough money to support him after the age of sixty-five.

If so heavy a burden is to be borne by a comparatively small number of hours, the business conduct of each hour must receive the most intensive cultivation. It is easy to overlook this because of pressure of other duties, from the natural hesitation which is felt in entering upon an untried field where difficulties lie, or because one has a feeling that all this is unnecessary, and that somehow matters will come out all right. Matters left to conduct themselves do "come out," as our old age pension funds within the profession testify. This attitude is pleasingly referred to by our French friends as "laissez-faire," which, in western slang means "let her rip!"

It may be accepted as certain that unless the dentist at thirty-five is willing to enter upon the intelligent and intensive cultivation of his twenty thousand hours, the chances of his acquiring a surplus by the age of fifty-five are very small. The dentist who decides against such cultivation or who omits it without decision, might well follow the example of a southern dentist. Soon after these articles were begun (in 1909) a letter was received, reading about as follows: "I am very glad to get the *Digest*. After reading the last number I saw why I had never made any money in dentistry and am not likely to ever make any. I talked to my father. He offered me a job at twenty-five dollars per week. I sold my office and took it. I feel better already."

The plan for cultivation of the income hours is short, simple and difficult to follow. It is no plan for a lazy man or a weakling. It is to carefully record all office expenses, to set a proper remuneration, to add to these at least one thousand dollars per year for the old age surplus; to divide the sum of these items by one thousand; to see that the minimum fees are at least equal to the amount obtained by the division; to collect these fees; to keep a system of books which will show a profit and loss account; to live within the established remuneration; to unswervingly save the surplus and, avoiding all wild-cat schemes, invest the money in securities approved by a reputable banker.

Not an easy program; not one to be dashed off in a moment of inspiration, but one demanding intelligence, patience and application. Dentistry as a livelihood is a business. If the dentist is to be a business director, a cost and credit man, an operator and a collector, he has no right to expect an easy task. If he operated a store, he would have all these difficulties and many others, and inattention would insure failure.

We, as a profession, have been blissfully unconscious of the necessity of any such program. Some have jeered at it in the past, some ridicule it today. Thousands will pass it by because of its difficulty. One phase of the result of this attitude can be told in a single sentence, as follows:

There are today, in one city of the United States, five hundred dentists old enough to be in good practice. who have proven by their conduct that they are unworthy of financial credit, who are living in straitened circumstances, and who have no hope of a competence in old age.—*Dental Digest.*

